# Supplementary material for: Can Pharmaceutical Excipients Threaten the Aquatic Environment? A Risk Assessment Based on the Microtox® Biotest
Source: Molecules. 2023 Sep 13;28(18):6590. doi: 10.3390/molecules28186590 (PMC10535389; doi:10.3390/molecules28186590)
Supplement: Supplementary file 1 [file molecules-28-06590-s001.zip › molecules-2580186-supplementary.pdf]

SUPPLEMENTARY MATERIAL

Can pharmaceutical excipients threaten the aquatic environment? – a risk assessment based on the Microtox® biotest

Turek, M.*[1], Różycka-Sokołowska, E.[1], Koprowski, M.[2], Marciniak, B.[1], Bałczewski, P.*[1,2]

[1] *Institute of Chemistry, Faculty of Science and Technology, Jan Długosz University in Częstochowa, Armii Krajowej 13/15, Częstochowa, 42-201, Poland*

[2] *Division of Organic Chemistry, Centre of Molecular and Macromolecular Studies, Polish Academy of Sciences, Sienkiewicza 112, Łódź, 90-363, Poland*

* *Corresponding authors e-mail addresses: pbalczew@cbmm.lodz.pl (P. Bałczewski), m.turek@ujd.edu.pl (M. Turek)*

Table S1. pH values of ARBs samples, excipients and mixtures used in Microtox® test.

ARB/excipient	pH
VAL	4.970
LOS-K	7.750
TEL	8.036
Microcrystalline cellulose (type PH102)	6.722
Sodium lauryl sulfate (SLS)	6.779
Magnesium carbonate (heavy)	10.257
Sodium starch glycolate (type A)	6.183
Colloidal anhydrous silica (Aerosil R200)	4.421
Povidone K25	3.889
Povidone K30	3.607
Croscarmellose sodium	4.347
Crosspovidone (type A)	6.045
Meglumine	10.830
Talc	9.082
Lactose monohydrate	5.113
Mannitol	6.390
Pregelatinized corn starch	5.432
Silica, nanopowder	4.681
Colloidal silica (LUDOX SM-30)	9.172
Sorbitol	7.446

Mixture	pH
VAL + microcrystalline cellulose	4.256
VAL + magnesium carbonate	7.408
VAL + povidone K25	3.990
VAL + sodium lauryl sulfate	3.961
VAL + colloidal silica	4.298
Magnesium carbonate + povidone K25	9.559
Magnesium carbonate + colloidal silica	9.231
Povidone K25 + colloidal silica	3.919
Povidone K25 + colloidal silica + magnesium carbonate	9.061
VAL + povidone K25 + colloidal silica	4.246
LOS-K + microcrystalline cellulose	7.101
LOS-K + mannitol	6.980
LOS-K + colloidal silica	7.405
LOS-K + talc	6.987
TEL + mannitol	7.007
TEL + povidone K25	5.450
TEL + meglumine	10.180
Mannitol + povidone K25	6.373
Mannitol + meglumine	10.493
Meglumine + povidone K25	10.161
VAL excipients	9.718
VAL + VAL excipients	5.157
LOS-K excipients	7.433
LOS-K + LOS-K excipients	7.509
TEL excipients	9.897
TEL + TEL excipients	10.033

Table S2. EC_{50} values of ARBs towards *A. fischeri* (modified Microtox® Basic Solid Phase Test) after 5, 15 and 30 minutes of incubation.

**ARB**	**EC_{50} [mg/L]**		
	**5 min**	**15 min**	**30 min**
**VAL**	143.20 [a]	234.83 [a]	150.5
**LOS-K**	583.88 [a]	659.02 [a]	276.7
**TEL**	196.52 [a]	99.70 [a]	77.31

[a)] – data published in *M. Turek, E. Różycka-Sokołowska, K. Owsianik, B. Marciniak, P. Bałczewski, J. Haz. Mat. 399 (2020) 122839*

Table S3. Parameters of response-dose equations (obtained by using eq. (2), manuscript) for pharmaceuticals mixtures (toxicity predicted by the IA model).

$E_{mix} = a \cdot \ln(c_{mix}) + b$			
**Mixture**	**$EC_{50,\ 30\ min}$**		
	**a**	**b**	**R^2**
VAL + microcrystalline cellulose	0.1816	-0.3603	0.98
VAL + magnesium carbonate	0.1193	0.1653	0.93
VAL + povidone K25	0.2298	-0.6985	0.96
VAL + sodium lauryl sulfate	0.0736	0.7035	0.87
VAL + colloidal silica	0.2236	-0.5748	0.99
Magnesium carbonate + povidone K25	0.1729	-0.1947	0.98
Magnesium carbonate + colloidal silica	0.1176	0.1594	0.99
Povidone K25 + colloidal silica	0.2562	-1.1072	0.99
Povidone K25 + colloidal silica + magnesium carbonate	0.1784	-0.1751	0.95
VAL + povidone K25 + colloidal silica	0.3348	-1.1889	0.95
LOS-K + microcrystalline cellulose	0.1630	-0.3345	0.97
LOS-K + mannitol	0.1687	-0.3917	0.99
LOS-K + colloidal silica	0.1552	-0.2710	0.97
LOS-K + talc	0.1163	0.0532	0.95
TEL + mannitol	0.0316	0.4778	1.00
TEL + povidone K25	0.0857	0.1425	1.00
TEL + meglumine	0.0745	0.5460	1.00
Mannitol + povidone K25	0.2221	-0.9890	1.00
Mannitol + meglumine	0.1565	0.0479	1.00
Meglumine + povidone K25	0.2166	-0.3081	0.97
